# Investigating the effect of forestry on leaf-litter arthropods (Algonquin Park, Ontario, Canada)

**DOI:** 10.1371/journal.pone.0178568

**Published:** 2017-06-02

**Authors:** M. Alex Smith, Amanda Boyd, Amelia Chan, Simonne Clout, Paulson des Brisay, Sarah Dolson, Thanushi Eagalle, Sean Espinola, Aaron Fairweather, Sydney Frank, Christopher Fruetel, Cristina Garrido Cortes, James Hall, Chris Ho, Eryk Matczak, Sandra McCubbin, Megan McPhee, Kate A. Pare, Kelsie Paris, Ellen Richard, Morgan Roblin, Cassandra Russell, Ryan Snyder, Carolyn Trombley, Tyler Schmitt, Caitlin Vandermeer, Connor Warne, Natasha Welch, Chelsie Xavier-Blower

**Affiliations:** Department of Integrative Biology, University of Guelph, Guelph, Ontario, Canada; Technical University in Zvolen, SLOVAKIA

## Abstract

Arthropods are the most diverse taxonomic group of terrestrial eukaryotes and are sensitive to physical alterations in their environment such as those caused by forestry. With their enormous diversity and physical omnipresence, arthropods could be powerful indicators of the effects of disturbance following forestry. When arthropods have been used to measure the effects of disturbance, the total diversity of some groups is often found to increase following forestry. However, these findings are frequently derived using a coarse taxonomic grain (family or order) to accommodate for various taxonomic impediments (including cryptic diversity and poorly resourced taxonomists). Our intent with this work was to determine the diversity of arthropods in and around Algonquin Park, and how this diversity was influenced by disturbance (in this case, forestry within the past 25 years). We used DNA barcode-derived diversity estimates (Barcode Index Number (BIN) richness) to avoid taxonomic impediments and as a source of genetic information with which we could conduct phylogenetic estimates of diversity (PD). Diversity patterns elucidated with PD are often, but not always congruent with taxonomic estimates–and departures from these expectations can help clarify disturbance effects that are hidden from richness studies alone. We found that BIN richness and PD were greater in disturbed (forested) areas, however when we controlled for the expected relationship between PD and BIN richness, we found that cut sites contained less PD than expected and that this diversity was more phylogenetically clustered than would be predicted by taxonomic richness. While disturbance may cause an evident increase in diversity, this diversity may not reflect the full evolutionary history of the assemblage within that area and thus a subtle effect of disturbance can be found decades following forestry.

## Introduction

Anthropogenic disturbance and fragmentation of natural habitats, such as forestry, can affect biodiversity [1, 2]. Previous studies have shown that forestry reduced the available natural habitat, which reduced beetle (Coleoptera) and oribatid mite (Acari) functional diversity and abundance [3–6]. Furthermore, Maraun et al. [7] observed a decline in oribatid mite and Collembola abundance after mechanical soil disturbance and removal of dead trees. Non-native species can also be affected by forestry and may further alter biodiversity [8, 9], as they can outcompete native species, change the natural ecology of the area, and are thought to have a homogenizing effect on the environment [3].

Forestry can alter microhabitats and the resources which terrestrial arthropods depend upon can be altered or lost, which can in turn influence important forest ecosystem processes [10, 11]. While forestry practices in Ontario and Canada have shifted towards selectively logging forests instead of using clear cutting methods, selective logging can still disturb and change the composition of the forest with poorly understood effects on the arthropod communities within [10]. For example, within the ants, forestry has been associated with both negative [9, 12] and positive [13, 14] effects on diversity.

Our understanding of the effects of forestry on some of the large, mobile vertebrates is well developed [10, 15, 16], while the effects of forestry on arthropod diversity are harder to untangle. For example, several researchers have noted a significant effect of forestry on spider diversity within the first decade following harvest [17], but that the spider community returns to the pre-cut assemblage after 30 years [18]. The abundance of several invertebrate groups (carabid beetles, collembolans and snails) was positively affected by strip clear-cuts compared to adjacent undisturbed areas [19]. Carabid diversity has been shown to be positively affected by clear cutting [20]. Mite diversity was found to be unaffected by clear-cutting, although abundance increased [6].

How forestry affects arthropod diversity has been examined specifically within Algonquin Park. For example, Simard and Fryxell [10], found that arthropod diversity (measured at the ordinal and family level) was greater in protected forests than in cut forests. Two studies [21, 22] found that for two groups of pollinators, species diversity was greater in cut forests than uncut or ‘wilderness’ areas of Algonquin. Examinations of Algonquin carabid beetles, 15–20 years post forestry [23], found an increase in generalist and open-habitat species. Thus, while incidences of both positive and negative effects of forestry exist for arthropod diversity, the specific effects of forestry on species-level diversity within most groups of arthropods remain unknown–frequently due to the difficulty in generating species-level taxonomic identifications for many groups [24, 25].

Many methods can be used to describe community diversity and structure; species richness describes the total number of species present, while phylogenetic estimates of diversity take into account how closely related species are to one another [26]. Phylogenetically clustered communities, include species which are more closely related than would be expected by chance. If the traits conferring the capacity to deal with an abiotic filter are phylogenetically conserved, this may suggest that the environment is having a filtering effect on individuals in the community and that only species with those specific traits are able to live in the area [26]. Various measures have been proposed to test the distribution of this phylogenetic diversity within communities. The oldest measure, phylogenetic diversity (PD) represents the sum of all branch lengths separating taxa in a community; PD increases as the taxon in the assemblage are more distantly related [27]. The degree of clustering shown by an assemblage can be estimated via the Nearest Taxon Index (NTI) which is calculated as 1- the mean nearest taxon distance (MNTD—the average branch length distance between sampled taxa). A positive NTI value that falls beneath 95% of the values from a randomised null model of phylogeny and/or community (e.g. a p-value of <0.95) indicates clustering, while negative NTI values with a p-value >0.95 indicate evenness [28]. Another measure of diversity is Faith’s phylogenetic diversity which is measured as sum of all phylogenetic branches in a community with the root from a larger regional phylogeny.

In this project, we set out to investigate which arthropod taxa characterise the forested and unforested areas in and around Algonquin Provincial Park, Ontario and whether these areas exhibit significant differences in measures of biotic diversity. If the disturbance history was the predominant force on the contemporary community, we would expect estimates of beta diversity to show high similarity between disturbed sites despite larger geographic distances between them. We used DNA barcodes as provisional species identifications to circumvent the “taxonomic impediments” caused by both resource shortage and cryptic diversity [24, 25] that plague many arthropod groups–particularly as our capacity to identify them is affected by sex [29] or life-history stage [30]. We hypothesised that if the forested areas were characterised by higher temperatures then they ought to have higher arthropod diversity. Furthermore, we used the DNA barcode information to compare richness estimates of diversity (the Barcode Index Number–BIN [31]), with phylogenetic estimates of alpha and beta diversity. While we expected PD and BIN richness to be correlated, departures from this correlation might suggest cases where there are different evolutionary histories resident in certain forests than would be predicted by taxonomic richness.

## Materials & methods

### Collection localities

**Fig 1:**
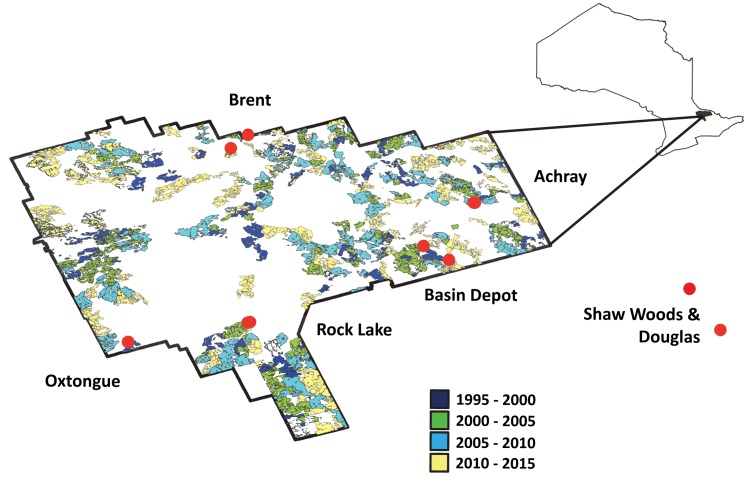
Map of Algonquin Provincial Park within Ontario, and the collection sites (red). Age categories of cut locations are illustrated here.

Collection localities were selected in and around Algonquin Provincial Park, Ontario (Fig 1). Within the Park we selected five paired sites that included an area that had been forested in the past 25 years (“cut”), and one nearby area that had not been forested (“uncut”) [32]. One pair of cut and uncut sites outside the park to the east of the park in Renfrew County was also included.

Algonquin Provincial Park is a large (7,360 km^2^) protected area in the eastern part of Ontario, Canada. The park is the oldest in the province (established in 1893) and has a unique history and contemporary mandate within Ontario given its concomitant emphasis on the protection of significant natural, cultural and recreational resources, as well as sustainable resource management for economic benefits [33]. The eastern portion of the park is predominantly pine forests (white pine, *Pinus strobus*, red pine, *Pinus resinosus*, and some jack pine, *Pinus banksiana*) while the western two-thirds of the park are deciduous (sugar maple, *Acer saccharum*, American beech, *Fagus grandifolia*, yellow birch, *Betula alleghaniensis*, and hemlock, *Tsuga canadensis*) [16]. Harvesting within the park is controlled by the Algonquin Forestry Authority [34]; areas designated for harvest are given a five-year period in which to be harvested, to account for weather, market, wildlife management, equipment availability, and other constraints to the process, and are then allowed to regenerate through planting and natural succession [32]. Contemporary forestry in the park is dominated by (in decreasing order of area or importance): selection, uniform shelterwood and clearcutting [32]. The park is home to a wide variety of species with the diversity of vascular plants and vertebrates estimated to be 1,000 and 200 respectively [33]. While the total insect diversity of Algonquin Park remains unknown, it has been estimated to be as high as 7,000 species [35].

### Field sampling protocol

Sampling within Algonquin Park was conducted in the summers of 2011 (June) and 2012 (June, July). Specimens were collected using a standardised protocol that was designed to focus principally on small arthropods in both the leaf-litter and above ground communities. Permission to collect within the park was issued by the Ontario Ministry of Natural Resources and Forestry (OMNRF). Traps included Malaise traps (canopy and terrestrial), yellow-pan traps, pitfall traps, leaf-litter sifting, mini-Winkler traps, and standardised active searching. In the cut and uncut areas of each forest samples were collected along a 50 m transect. Along each transect at 10m intervals six collections were made consisting of: pitfall traps, yellow pan traps, a 1 m² leaf-litter collection (actively sifted), mini-Winkler (sifted for 3 days), and a terrestrial and canopy Malaise trap maintained for a one week period. Furthermore, active searching was conducted for an hour during trap deployment and collection. Full methods were derived from [36], and can be examined in the supplemental information (http://www.ecography.org/sites/ecography.org/files/appendix/ecog-00631.pdf). Collected specimens were stored in 90% ethanol at 4°C and then morphologically sorted to ordinal level (“Ordinal lots”).

Sampling at the site pair outside the boundaries of Algonquin Park was conducted exclusively using terrestrial Townes-style Malaise traps [37] between 2010 and 2012 throughout the flight season (~April–November). The uncut site (the Shaw Woods), is one of the few remaining sections of first growth deciduous forest (www.shawwoods.ca/) [38, 39], while the cut site (Douglas) is a privately owned wood lot. The owners of each location gave permission to conduct the study on these sites. The distances between all sites was less than 80 km on average and between specific paired sites was less than 5 km (max 11 km, min 90 m, S1 Table).

Within the five site pairs of Algonquin Park there were 7 sampling days in 2011 and 14 in 2012 for six collection methods. For the site pair in Renfrew County there was 180 sampling days in 2011 and 2012 based exclusively on terrestrial Malaise traps. For all sites, we sampled for a combined total of 486 site days.

### Air temperature

In the spring of 2015, HOBO Pendant® Temperature 64K Data Loggers were placed at the approximate location of the Malaise trap at each site approximately 50 cm above the ground and set to record temperature every 10 minutes. These data loggers were collected in the winter and spring of 2016.

### GigaPan habitat panoramas

At all collection localities we used a GigaPan robot to take thousands of photographs to stitch together into high-resolution panoramic photographs for each site as a method of capturing the details of these locations (e.g. S1 Fig) [36, 40].

### DNA protocols

**Specimen selection for barcoding.** The overwhelming diversity and abundance of individuals collected with our sampling protocol necessitated decisions regarding which taxa would be barcoded and analysed at a species-level. We selected the taxa that included the most abundant samples in the Malaise traps (Diptera, Hymenoptera, Diplopoda, Collembola, Araneae, Hemiptera, Coleoptera, Lepidoptera and Formicidae). Within these taxa, a subset of specimens was selected for DNA barcoding by one of two methods. Within the Hemiptera, Coleoptera, Formicidae and Collembola, specimens were first sorted to morphospecies by non-taxonomic expert student co-authors [41] from which representative members were sequenced. Alternatively, within the Lepidoptera, Hymenoptera, Diptera, Diplopoda and Araneae specimens we selected samples for DNA barcoding by randomly sampling specimens across sites within that taxon.Following sample selection and sub-sampling from the collection lot or ordinal lot, specimens were photographed using a Leica Z16 APOA Macroscope using the Leica Application Suite V4.3, and photographs were uploaded to the Barcode of Life Data System ([42] BOLD; www.barcodinglife.org). Single legs were sampled from the selected individuals for DNA extraction and subsequent amplification of the DNA barcode region.

#### Barcoding

We analyzed DNA barcodes (the 5’ region of the cytochrome *c* oxidase I (CO1)) gene for the arthropod specimens from the nine taxa we collected in and around Algonquin Park in 2011 and 2012. DNA barcodes were amplified from total DNA extracts that had been prepared from single legs (for most groups, whole specimens for Collembola) using a standard glass fibre protocol [43]. This 658 bp region near the 5’ terminus of the COI gene was then generated using standard primers following established protocols [44–46].

Using these DNA barcodes, we calculated alpha and beta diversity estimates using both BINs and phylogenetic trees from which we calculated measures of phylogenetic diversity [27]. We used BINs as a measure of taxon richness in order to determine the impact of forestry on arthropod diversity. While we do not expect there to be 100% congruence between BINS and formally described species, for many groups where the agreement has been examined there has been a close relationship between the two. Departures from a one to one relationship between the identification of a BIN and a species might occur through various reasons that include mitochondrial introgression and heteroplasmy [47], *Wolbachia* infection [48] and the existence cryptic species existing within formally described names [44]. However, for the taxa we have examined here, DNA barcodes have previously shown their utility as a species proxy with generally high interspecific sequence variation, low intraspecific variation and high congruence between BINS and formally named species. Specifically, we refer to previous comparisons made with the taxa examined here where the degree of congruence between DNA barcode BINS or divergence with species identified using traditional means ranged between 91.5% (Hemiptera [49]), 95% (millipedes [50]), 96% (ants [51]), 97% (moths [52]) 98.3% (beetles [53])and 98.9% (spiders ([54]). In hyperdiverse groups, such as the Diptera, where one to one comparisons have not yet been made, others have found that expected species counts and BIN counts approach 1:1 when examined on a national level [24, 55]. Finally, in groups, such as the Collembola and parasitoid wasps where previous taxonomy is expected to lag behind actual diversity due to various taxonomic impediments [25], DNA barcode BINS have been found to be useful species-proxies in the absence of formalized taxonomic identifications [56, 57]. Based on such a high degree of congruence, we here use BIN identifications as a proxy for traditional names. The data associated with our analyses are entirely contained within the Barcode of Life Data System (BOLD [42]) and as names are associated with each record; this transition from provisional molecular name to formal identification will be recorded in this database.

Measures of PD were calculated from a phylogeny constructed using the highest quality sequence (longest read length and smallest number of ambiguities) from each BIN using a Maximum Likelihood tree based on the General Time Reversible model (GTR) [58]. The GTR was selected due to the lowest BIC scores (Bayesian Information Criterion) of the Maximum Likelihood fits of 24 different nucleotide substitution models run in MEGA6 [59]. Measures of phylogenetic diversity (PD), phylogenetic community structure (Nearest taxon index (NTI)–[60]) and phylobetadiversity (the mean nearest taxon distance—an estimate of the phylogenetic distance between pairs of species drawn from two sites (comdistnt) and thus a phylogenetic measure of betadiversity) were calculated using the picante package [61] in R (R Core Team).

To examine elements of PD not explained by BIN richness, we controlled for the expected relationship between BINS and PD by linear regression and then used the residuals from this relationship to compare cut and uncut sites. We asked whether there was more or less phylogenetic diversity in these treatments than predicted by the species diversity.

Alpha diversity (taxon or BIN richness) and beta diversity (Jaccard Index; [62]) were calculated based on incidence matrices for each of the nine taxa using EstimateS (v 9.1; [63]).

### Abundance

For the Diptera and the Myriapoda we calculated specimen abundance and family level diversity between cut and uncut sites for the collections made in 2012 for the 10 sites within Algonquin Park.

## Results

### Temperatures

**Fig 2:**
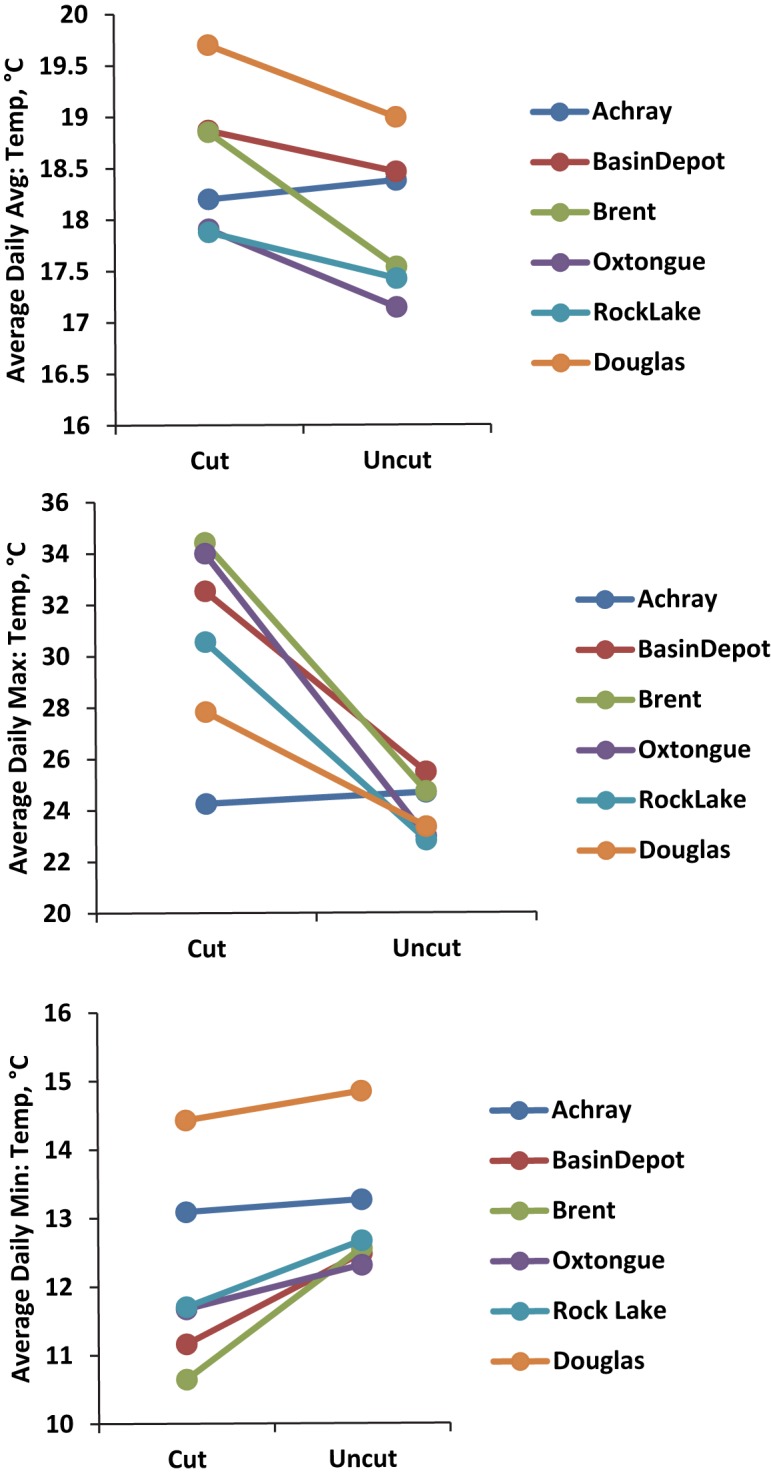
Temperature (max, min and average) calculated at each of the six paired locations for three months in 2015. With one site-pair exception (Achray), cut sites displayed higher average and maximum daily temperatures and also lower daily minimum temperatures.

Cut sites were characterised by higher daily average and daily maximum temperatures and lower daily minimum temperatures (Fig 2, S2 Fig, S1 Table). In addition, cut sites exhibited much larger fluctuations in observed daily maximum temperatures. There was one exception to this at the Achray site where maximum and average temperatures were not significantly higher but lower, between cut and uncut sites.

### Diversity

From the original field collections, we sorted 3,415 ordinal lots (often containing multiple individuals) and sequenced 4,450 individuals from nine orders. Restricting our analyses to only these 9 orders still included a significant component of the total diversity–more than 3,000 ordinal lots representing 60% of the total collections (S3 Fig). In our analyses, we chose to focus on the highest quality individual sequences (4,202). Because sampling efforts were uneven in time and collection type between the park site pairs and the pair outside the park, we maintained separate pairwise comparisons.

Specimen records, photographs and details for all DNA barcoded samples are available at the following public DOI: dx.doi.org/10.5883/DS-ASALGONQ.

We collected 1,746 BINS during the total 486 site days. Most of these BINS (1,240 or 71%) were only observed a single time in the dataset (S3C Fig). Despite the relatively close distance between each of these collection locations, there was limited overlap in the species found at cut and uncut sites. At the time of writing, there are 47 BINS from these collections that represent the only instance of that species in BOLD. When we used EstimateS to extrapolate from our observed diversity to what we would expect to see if we had sampled 1,000 sites–diversity was estimated to be just over 4,000 species (S4 Fig). The majority of species recovered were from Malaise traps and pitfalls, however, the diversity recovered via the nine different collection methods were substantially different (S5 Fig).

It is possible that our two sub-sampling protocols (randomly within sites and representatives of morphospecies within sites) might generate different relations between BIN and morphological species diversity. However, such patterns will not be directly testable until we have formal taxonomic names for the samples of each taxon. We do note that in each sampling protocol there was a strong and significant relationship between BINs (our species proxy) and PD. This suggests that our findings are likely resilient to any effect that a difference in sub-sampling protocol might produce.

The most frequently collected and sequenced species was a cranefly (Diptera; Limoniidae; *Limonia immatura)*. While no species was present at all 12 sites, there was one species of Parajulidae millipede (BOLD:ACM9079) present at 11 sites, two species at ten sites (the ant *Camponotus pennsylvanicus* and the Amaurobiidae spider *Callobius bennetti*) and three species present at nine sites (two ants *Lasius alienus* and *Camponotus herculeanus* and one spider *Pocadicnemis americana*). These widespread and frequently collected species are all species that are present throughout the Holarctic.

### Phylogenetic measures

**Fig 3:**
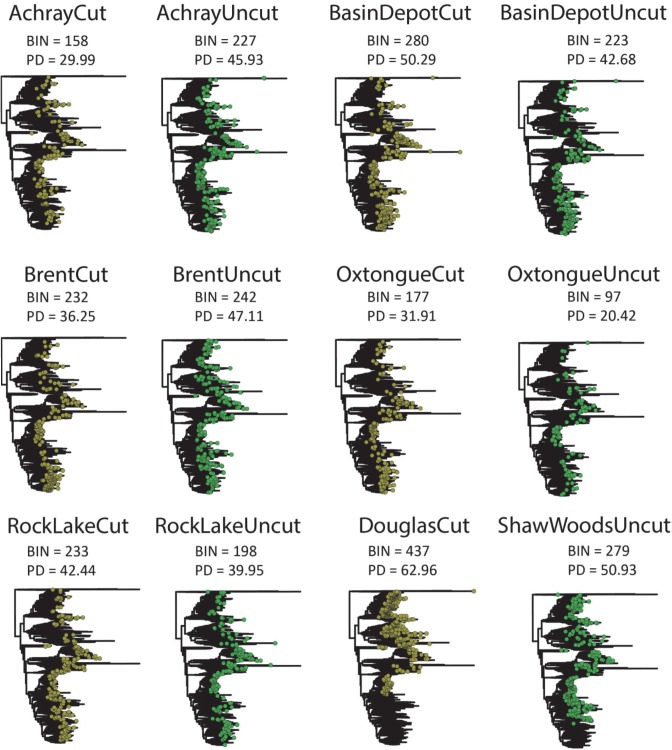
Maximum-likelihood tree of total diversity evident at each of the six paired sites (cut in brown and uncut in green). BIN richness and total phylogenetic diversity (PD) evident at each site is noted.

**Fig 4:**
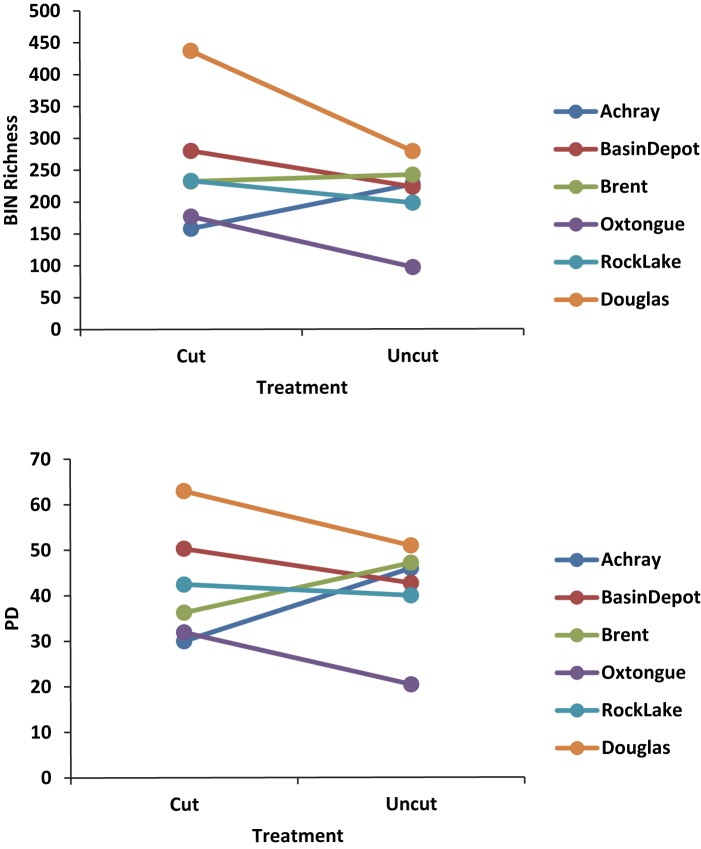
Pairwise comparisons between cut and uncut site pairs for BIN richness and phylogenetic diversity.

**Fig 5:**
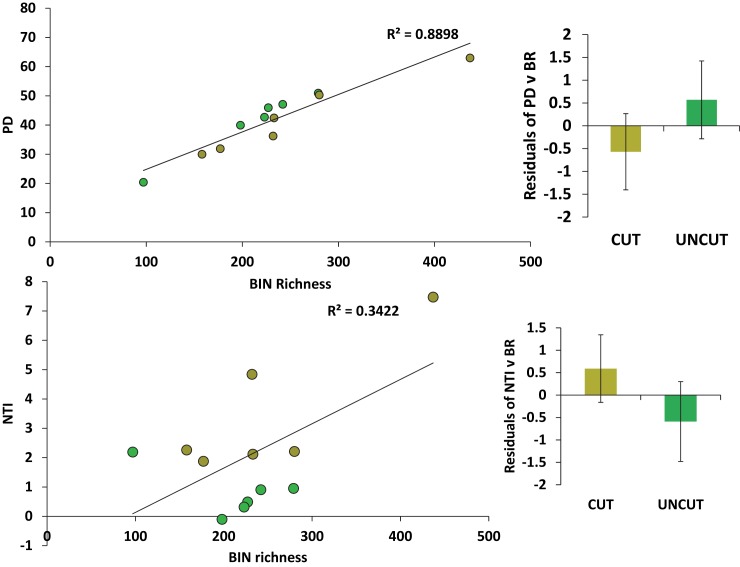
**The relationship between BIN Richness (BR) and Phylogenetic Diversity (PD) (A) and Nearest Taxon Index (NTI) (B).** Sites coded as cut (brown) or uncut (green). When the relationship between BR and PD is controlled for, the cut sites displayed less phylogenetic diversity that was more phylogenetically clustered than at uncut sites.

Two-thirds of the site pairs considered exhibited a decline in PD from cut to uncut (Figs 3 and 4, S1 Table). However, when we compared the residuals of PD and BIN richness we found that cut sites had less PD than predicted by BIN richness (Fig 5, S1 Table). Furthermore, nearly all (5/6) of the cut sites were found to be phylogenetically clustered (4 of these significantly) (Fig 5) and the cut sites were found to be more clustered than predicted by BIN richness (S1 Table). No sites were found to be significantly dispersed.

### Betadiversity

**Fig 6:**
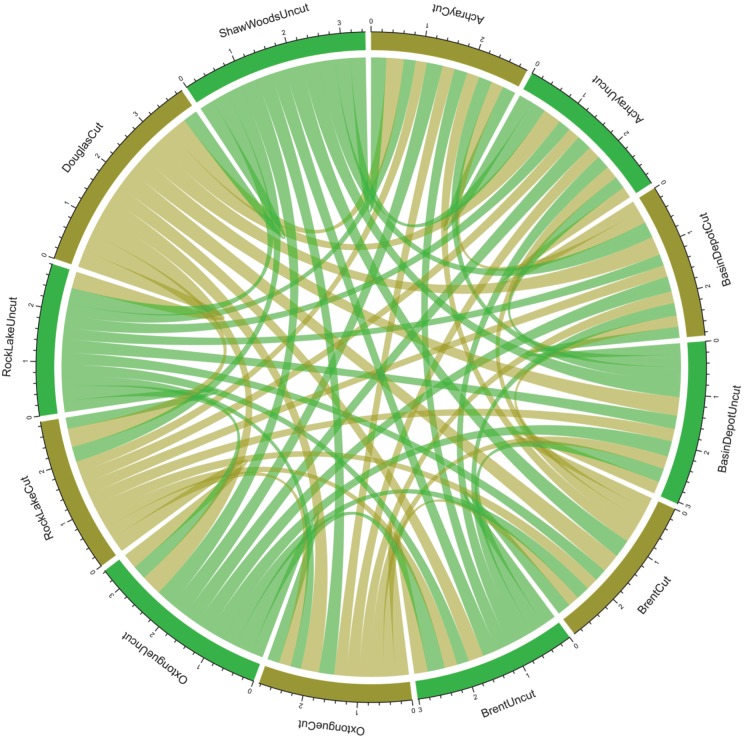
Chord diagram that illustrates the phylobetadiversity (site dissimilarity measured phylogenetically as the distance between pairs of species drawn from two sites (comdistnt)). Cut sites are in brown and uncut sites are in green.

**Fig 7:**
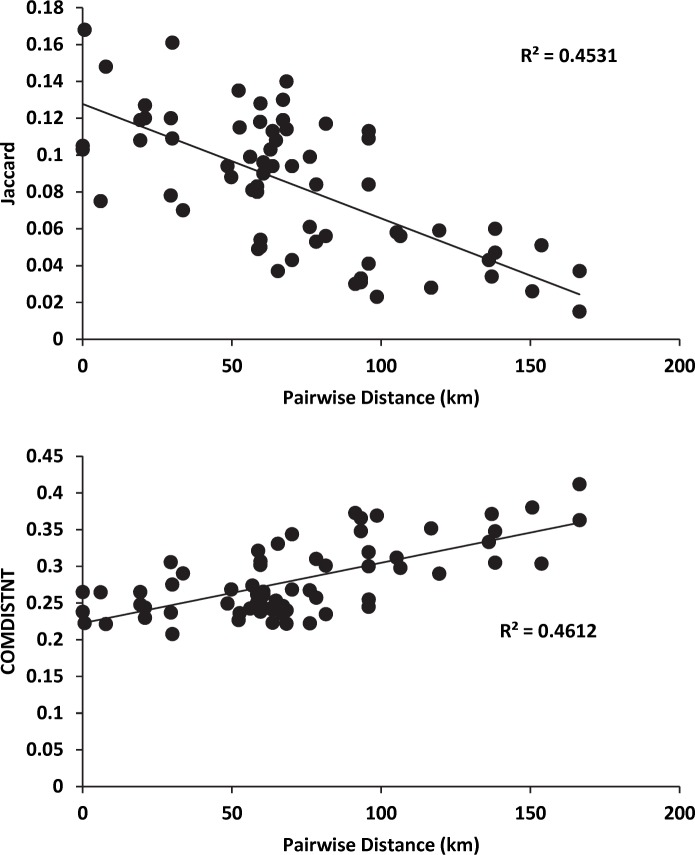
**Relationships between pairwise measures of betadiversity ((A) Jaccard and (B) phylobetadiversity) and geographic distance.** Note that similarity declines, even at the small distances evident here (<200 km). Also note the tighter relationship when betadiversity was estimated phylogenetically (B).

Overlap between sites was limited as measures of Jaccard (similarity) or phylobetadiversity (dissimilarity) were small and not predicted by treatment (cut or uncut) (Fig 6). However, even at the small distances evident here (<200 km) the betadiversity between each site (measured via BINS or phylogenetic measures) declined with distance (Fig 7).

### Abundance

There were a total of 24,725 dipteran specimens collected in 2012 and abundance at cut sites was significantly greater than in uncut sites (χ^2^ = 2350.70, df = 5, p< 0.001). There were 823 millipedes collected in 2011 and 2012 and the abundance at uncut sites was higher than cut sites, but not significantly different (χ^2^ = -1.21, df = 4, p< 0.15).

## Discussion

We found that the forests in and around Algonquin Park hosted a diverse assemblage of arthropods, many of which were new representatives of BINs to BOLD, despite living in an area that has been intensively sampled over the past decade. Using BIN-based estimates of taxonomic richness and phylogenetic estimates of diversity derived from these DNA barcode records, we found that although cut sites tended to have greater diversity there was no significant difference in the diversity between forested and paired unforested sites. However, when we controlled for the relationship between BIN richness and PD, there was less phylogenetic diversity and it was more phylogenetically clustered in the forested sites compared to unforested sites. This decoupling suggests that while the effects of forestry on these arthropod communities may be subtle (i.e. the pattern that cut sites contain less PD than expected compared to uncut sites is subtle compared to the overall variation in PD–clearly there are more elements influencing such patterns than forestry alone), such disturbances have had an enduring and potentially significant effect on the evolutionary history resident within these forests. Forest et al [64] also found that the PD scaled in a complex manner with taxon richness and some regions had more or less PD than would be expected from their richness alone. If the phylogenetic changes we documented here are coupled with functional changes, this suggests that there may be traits present in the forested sites that permit greater success in these disturbed environments. Future work ought to focus on testing for changes in functional traits (perhaps responses to heat or desiccation). Recently, [65] it was reported that disturbance in the form of agriculture produced significant alterations in phylogenetic and functional diversity without any change in the taxonomic richness of plants or butterflies.

Effect of forestry on diversity: Others have found an increase in diversity associated with forestry. For example Nol et al [21] found an increase in the abundance and diversity of flower-visiting flies, beetles and bees within forested areas of Algonquin Park compared to wilderness areas. However, one novel consideration of this relationship that is elucidated by our work is the lower than expected PD values for these cut sites. Essentially, while the cut sites have greater diversity than the uncut sites, the diversity that is present in these undisturbed habitats contains a larger than expected proportion of the evolutionary history of the species present (i.e. PD). Furthermore, we found more evidence of phylogenetic clustering in the cut sites compared to the undisturbed sites. Together, these findings suggest that while our initial impression of increased diversity is associated with forestry disturbance, there may be long-term costs associated with the disturbance if phylogenetic diversity is lost and clustered lineages associated with the new abiotic conditions on cut sites begin to predominate. The time-scale upon which we were able to sample does not allow us to determine if this effect would subside with time following disturbance, however, we would predict that sustained disturbance would lead to greater substantial changes in the invertebrate community. The decade-level times between disturbances currently provided for in the management plan (25 years) [32] likely provides sufficient time to prevent the runaway effect of this selection.

Whether betadiversity was measured using Jaccard (similarity) or phylobetadiversity (dissimilarity) we found small differences between sites that was not predicted by the cut or uncut nature of that site. Meanwhile, the betadiversity (measured via BINS or phylogenetic measures) declined with distance, even at the small scale examined here (<200 km). This suggests to us, that while the changes we identified in alpha diversity were forestry-associated, there was also a significant regional trend in beta diversity across the park where similarity declines across relatively short geographic distances. Furthermore, as in alpha diversity, this relationship was more tightly described when betadiversity was estimated phylogenetically.

Disturbance duration and intensity: We had initially imagined that it was possible that our sampling (broadly at a decadal level post disturbance in most cases) occurred too far past the disturbance event(s) for us to measure tangible effects today. However, we documented significant differences in diversity and community structure (when we controlled for taxonomic richness) between forested and unforested areas of Algonquin Park. Such subtle changes, evident multiple decades following the disturbance, mirrors some of the conclusions that Sutherland et al [66] reached studying old-growth forest stands on Vancouver Island. Here, they found complex trajectories for forest recovery following logging–including signals of disturbance 200+ years following the event.

Congruence of PD and BIN: Despite being forested within the past several decades, our forested sites were less diverse and more phylogenetically clustered than their paired, uncut sites would have predicted. We controlled for the expected high congruence between taxonomic or BIN richness and barcode-derived estimates of phylogenetic diversity and revealed a significant difference between the forested and unforested areas for both phylogenetic diversity (PD lower in cut sites) and phylogenetic community structure (NTI–higher (clustered) in cut sites). While the relationship between species richness (SR) and PD is expected to decline with an increasingly large species pool [67], the relationship we documented here was strong and significant. Our test of the residuals from SR:PD relationship revealed the treatment effect on diversity. While SR and PD are expected to be correlated, departures from this trend have been invoked by others to reveal patterns critical to conservation in other taxa. For example, Laity et al. [68] worked with multiple taxonomic groups and found that PD measures across Australia with a focus on two highly biodiverse regions, were greater than predicted by taxonomic richness. They suggested that this phylogenetic difference was important to conservation as it suggested the previously unappreciated potentially greater feature, or functional, diversity that a taxonomic measure alone would have missed. Also in Australia, Costion et al. [69] examined decoupled patterns of PD and genus richness (GR) for plants. They found that areas with positive PD:GR residuals contained multiple recent immigrant plant lineages, while negative PD residual areas were dominated by ancient relict lineages.

We found that disturbed communities tend to contain phylogenetically clustered species. If these closely related species also possess similar traits (that is, if phylogeny and function are coupled) this suggests the existence of a subtle filter for disturbance-tolerant taxa. Testing this connection is beyond the scope of this paper, but the existence of the phylogenetic pattern ought to direct future hypotheses and predictions. Phylogenetically clustered species within disturbed areas have been documented before. For example, Helmus et al [70] found that communities of freshwater zooplankton contained closely related species following disturbance. As we found here, their trend was prevalent independent of species richness.

The Effect of Canopy and Time: Canopy loss has distinct effects on the microenvironment [18]. We have shown here that such effects are evident even more than 20 years following the disturbance. We found that the forested sites were characterised by higher temperatures (particularly daily maximum temperatures), had greater diversity (via BIN richness or phylogenetic measures) and tended to be more phylogenetically clustered than unforested sites. The unforested sites were less diverse but tended to have more phylogenetic diversity than predicted by BIN richness. Canopy-mediated changes in heat and light have been associated with changes in the diversity and abundance of some arthropod groups–particularly pollinator groups. For example, Proctor et al [22] related the differential species diversity of syrphid flies and bees between cut forests and uncut controls to the relative increase in light caused by the harvest. They showed that the differences in abundance and diversity were not present during the spring, when the canopy was absent. Rutgers-Kelly and Richards [71] found that the abundance and diversity of bees increased for the first three years following disturbance and then declined afterwards. The presence of a filled canopy not only affects light (as tested by Proctor et al. [22]), but also the temperature variability experienced by the organisms living here. This is evident in the changes we documented between cut and uncut sites for maximum temperature (higher in cut sites) and minimum daily temperatures (lower in cut sites).

## Conclusions & next steps

One advantage of the approach we employed here, via BINS and phylogenetic measures of diversity, is that our biodiversity assessments are transparent and robust to taxonomic uncertainty and changes [72]. This is important, as many of the groups we have identified are beset by several impediments to taxonomy [24, 25] and so this style of analysis permitted us to complete our analyses in advance of completed formally described species, and also “sets the table” for the completion of such analyses by interested taxonomists in the future.

We demonstrated an apparent decoupling of phylogenetic diversity and taxonomic richness that should help our understanding of the effect of disturbance on protected areas. Interestingly, even in well sampled areas like Ontario, our intense sampling yielded new species-level discoveries. This included new discoveries to science (e.g. species of moth and parasitoid wasp described in S1 Text) but also BINS that were evidently new to the large publicly accessible library of DNA barcodes (spider, millipede and many ichneumonid wasps described in S1 Text). Such discoveries, and the use of the publicly accessible data in a phylogenetic context, ought to be supported in the maintenance of long-term sampling and research of even presumably well-known areas.

## Supporting information

S1 Fig GigaPan photographs were captured by setting up a camera attached to a small robot on a tripod at each site, approximately 30 cm above the forest floor. The robot was programmed to take a series of overlapping photos of the site, and these images were imported into the GigaPan stitching software (v 1.0.0805) and stitched together to form a single panoramic photograph. This figure is of the GigaPan panorama from Brent station (uncut) showing the detail evident within these high-resolution photographs (www.gigapan.com/gigapans/107071). The two insets are of two snapshots where an abundance of crane flies (Limoniidae) are evident. A public gallery of panoramas from all sites can be explored here http://www.gigapan.com/galleries/7832/gigapans.
(PDF)

S2 Fig Daily maximum temperature observed in each of the six sampling locations for three months of 2015. Forested locations (brown) displayed much larger fluctuations than uncut sites (green).
(PDF)

S3 Fig Summary collection details. (A) Total number of Ordinal lots collected from cut and uncut sites, (B) the number of sequences generated from each taxon compared to the number of lots and (C) the frequency that each species was collected as a proportion of the total. Most species were only collected a single time.
(PDF)

S4 Fig Site-based accumulation curve extrapolated to 100 sites. Following this extrapolation, the expected diversity after approaches an asymptote between 100 and 100 sites (After 100 sites estimated diversity was 4,277 species and after further expansion to 1,000 sites it was 4,362).
(PDF)

S5 Fig Most species were recovered from two trap types (Malaise and pitfall) while there were substantially different communities recovered from each trap type. (PDF)

S1 Table Temperature, diversity and community structure measures differ between treatments. Statistical comparisons (t-tests) between measures of temperature, NTI and PD across cut and uncut sites.
(PDF)

S1 Text Species Narratives: An example from each of the nine taxa examined here of species discovered in this work. The specific BINS and/or specimen accessions in these narratives are listed below and are all accessible via the public DOI: dx.doi.org/10.5883/DS-ASALGONQ. A) BOLD:AAG9100, B) BOLD:AAU8720, C) BOLD:AAA3898 and BOLD:AAM6820 D) BOLD:AAB2868 and BOLD:AAA4157, E) BOLD:AAU6930 (BIOUG06758-F09), F) BOLD:ADA0234, G) BOLD:AAJ2208 (BIOUG14581-A01), H) BOLD:ABY1771 (BIOUG02365-E07) and I) BOLD:ACA2347 (BIOUG02613-A01).
(DOCX)
