# Supplementary figures and images for: Investigating the effect of forestry on leaf-litter arthropods (Algonquin Park, Ontario, Canada)
Source: PLoS One. 2017 Jun 2;12(6):e0178568. doi: 10.1371/journal.pone.0178568 (PMC5456079; doi:10.1371/journal.pone.0178568)

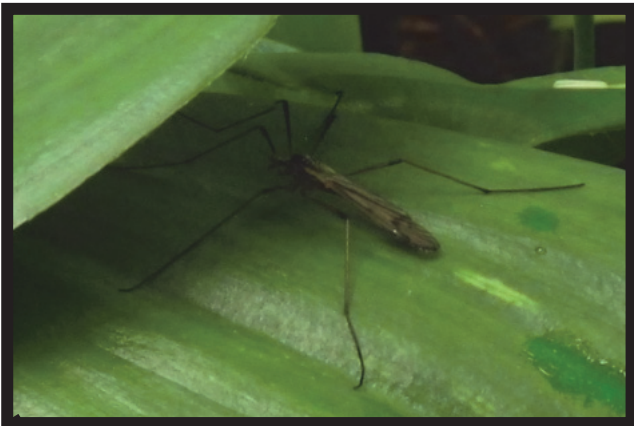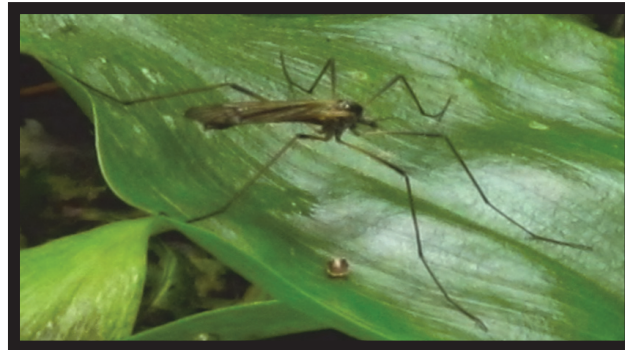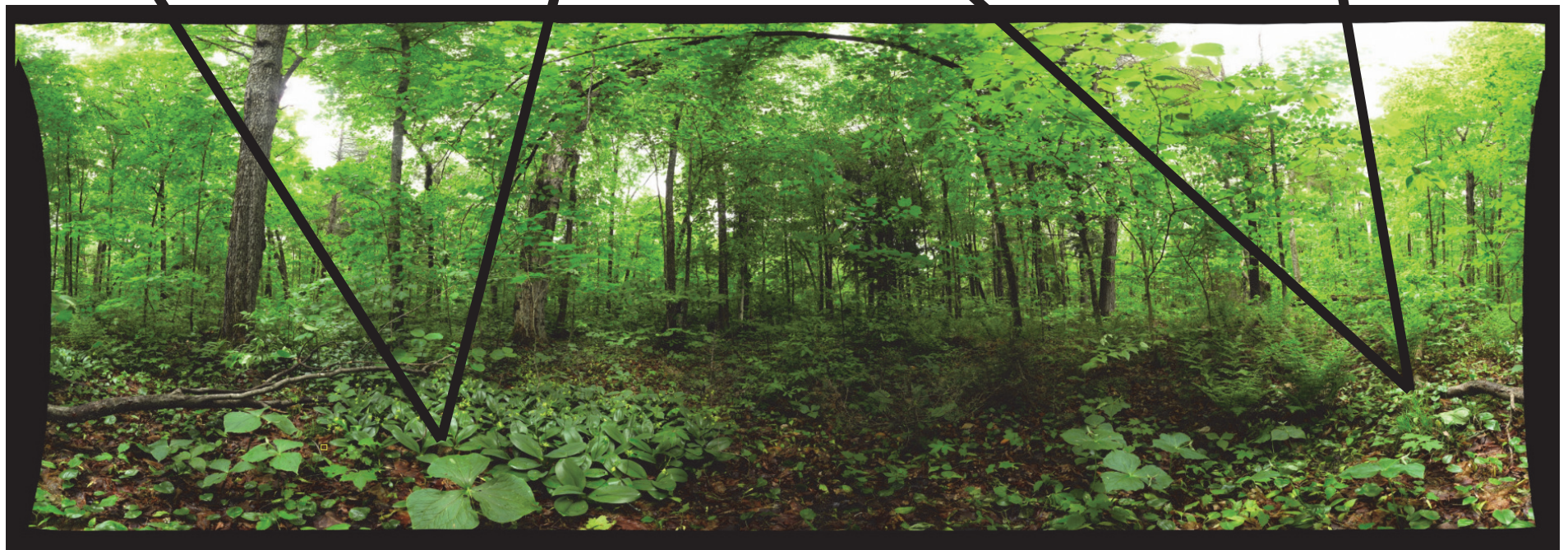

Supplement: S1 Fig — The robot was programmed to take a series of overlapping photos of the site, and these images were imported into the GigaPan stitching software (v 1.0.0805) and stitched together to form a single panoramic photograph. This figure is of the GigaPan panorama from Brent station (uncut) showing the detail evident within these high-resolution photographs (www.gigapan.com/gigapans/107071). The two insets are of two snapshots where an abundance of crane flies (Limoniidae) are evident. A public gallery of panoramas from all sites can be explored here http://www.gigapan.com/galleries/7832/gigapans. (PDF) [file pone.0178568.s001.pdf]

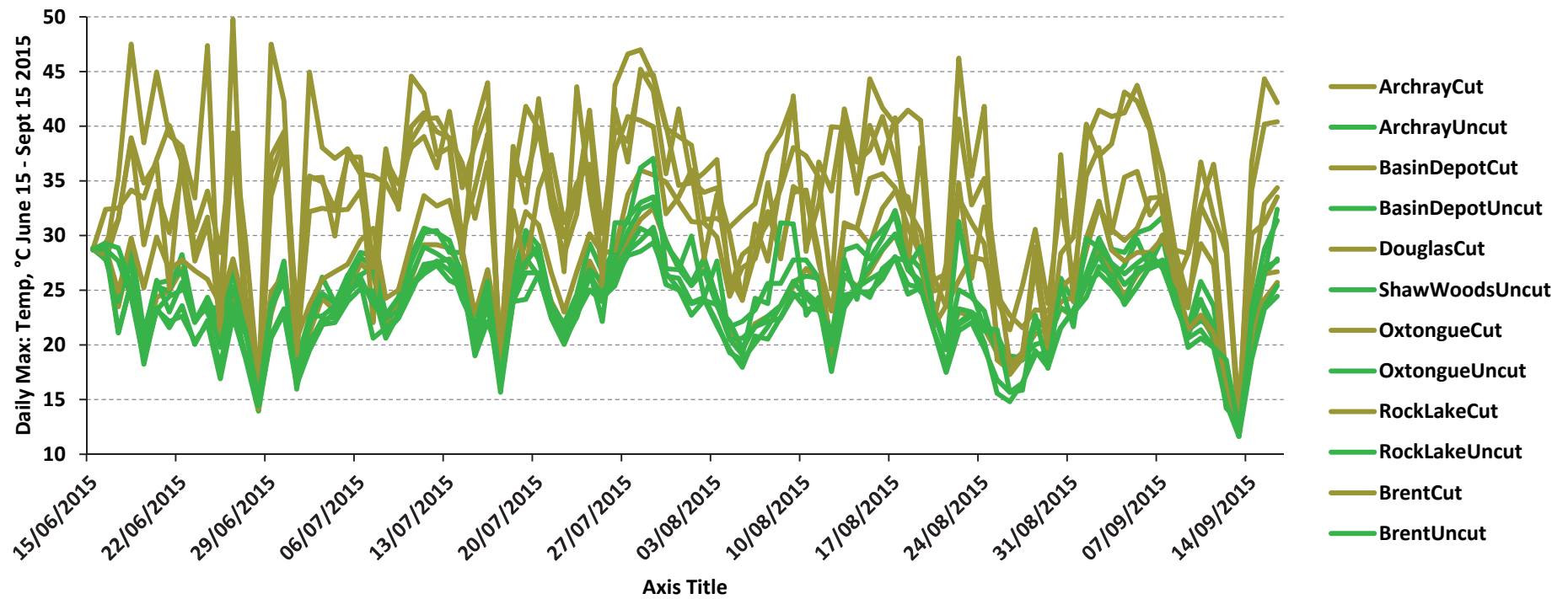

Supplement: S2 Fig — Forested locations (brown) displayed much larger fluctuations than uncut sites (green). (PDF) [file pone.0178568.s002.pdf]

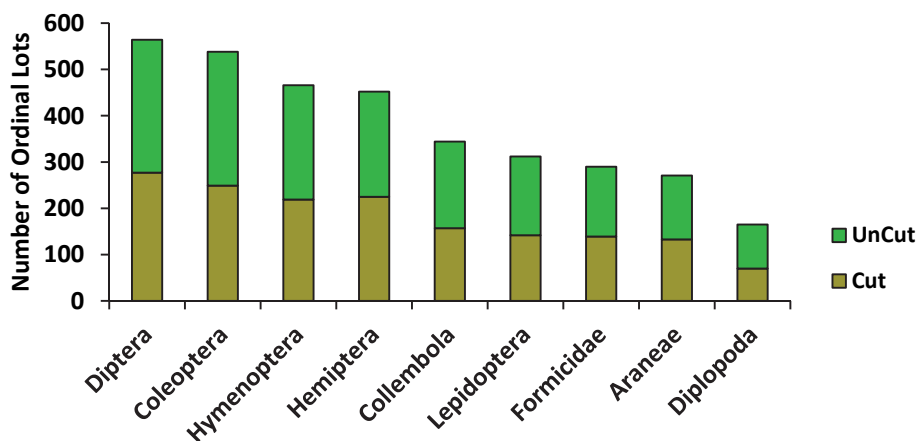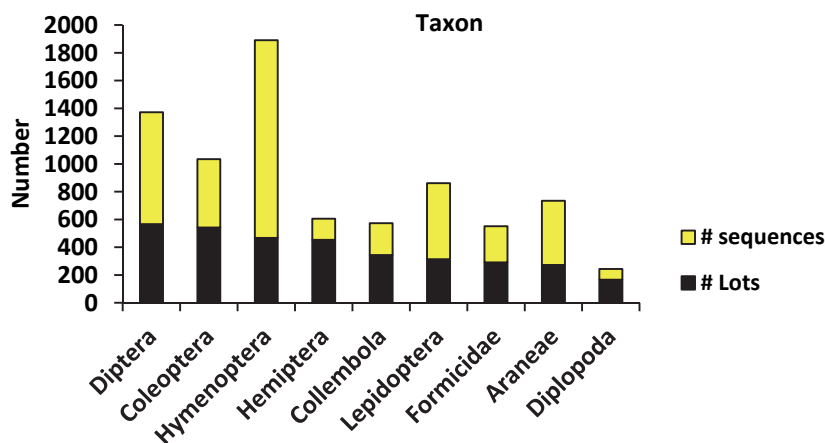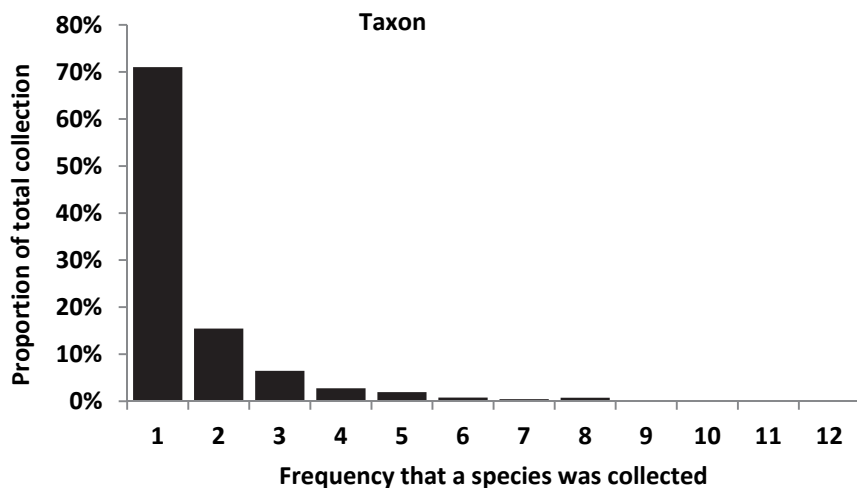

Supplement: S3 Fig — (A) Total number of Ordinal lots collected from cut and uncut sites, (B) the number of sequences generated from each taxon compared to the number of lots and (C) the frequency that each species was collected as a proportion of the total. Most species were only collected a single time. (PDF) [file pone.0178568.s003.pdf]

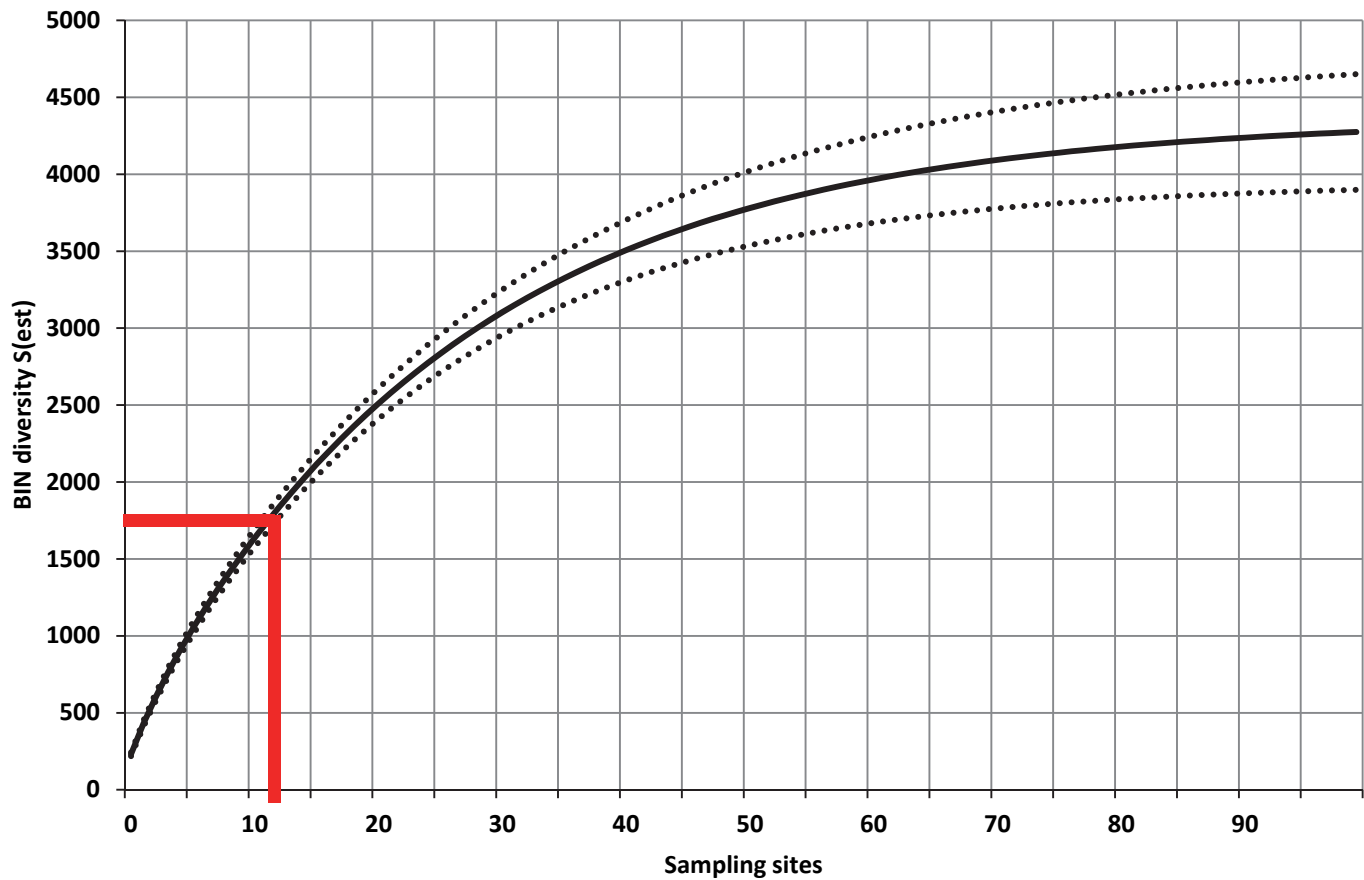

Supplement: S4 Fig — Following this extrapolation, the expected diversity after approaches an asymptote between 100 and 100 sites (After 100 sites estimated diversity was 4,277 species and after further expansion to 1,000 sites it was 4,362). (PDF) [file pone.0178568.s004.pdf]

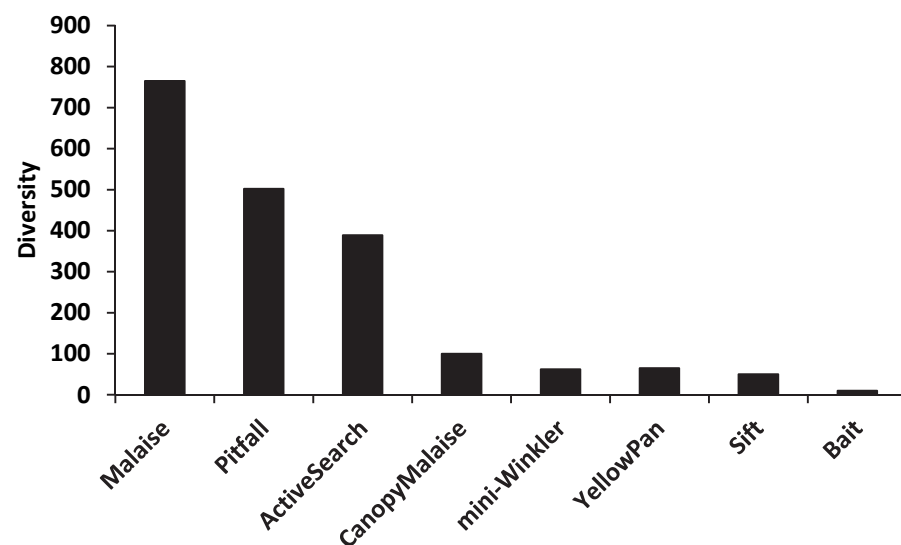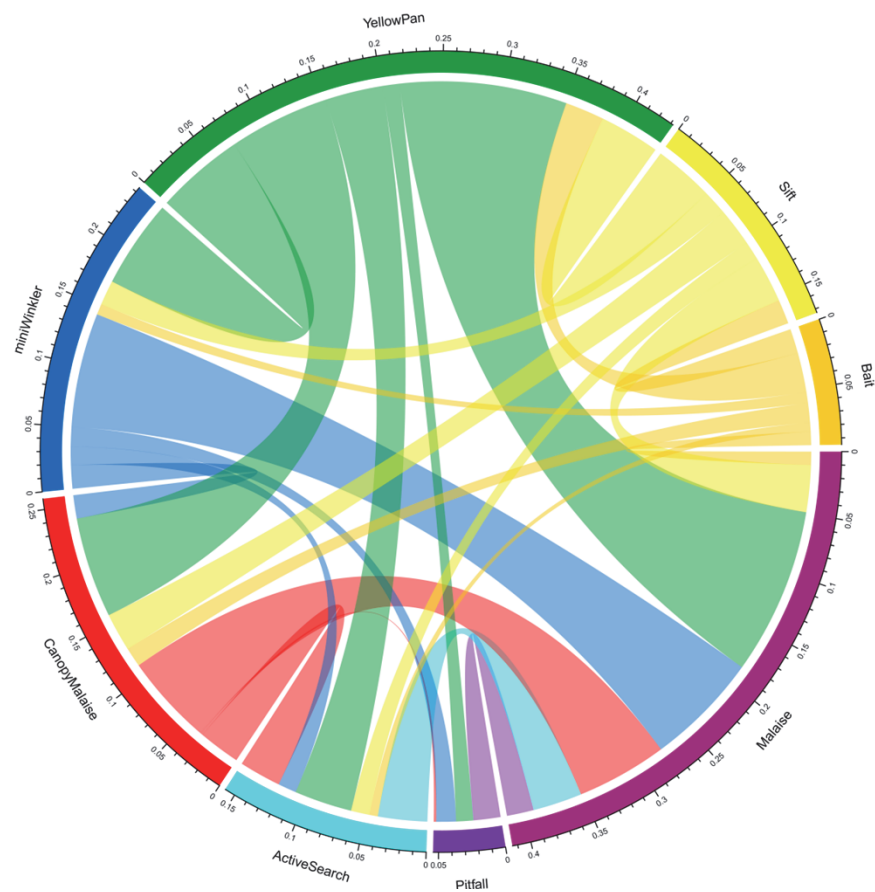

Supplement: S5 Fig — (PDF) [file pone.0178568.s005.pdf]
