# Supplementary material for: Investigating the effect of forestry on leaf-litter arthropods (Algonquin Park, Ontario, Canada)
Source: PLoS One. 2017 Jun 2;12(6):e0178568. doi: 10.1371/journal.pone.0178568 (PMC5456079; doi:10.1371/journal.pone.0178568)
Supplement: S1 Table — Statistical comparisons (t-tests) between measures of temperature, NTI and PD across cut and uncut sites. (PDF) [file pone.0178568.s006.pdf]

SI Table 1: Temperature, diversity and community structure measures differ between treatments. Statistical comparisons (t-tests) between measures of temperature, NTI and PD across cut and uncut sites.

t-tests between cut and uncut locations with p-values

	**MAX TEMP**		**MIN TEMP**		**AVG TEMP**	
	t-test value	p-value	t-test value	p-value	t-test value	p-value
**Archray**	-6.375	<0.001	-8.366	<0.001	-9.827	<0.001
**BasinDepot**	18.747	<0.001	-12.957	<0.001	4.517	<0.001
**Douglas**	19.687	<0.001	-4.994	<0.001	13.79	<0.001
**Oxtongue**	16.139	<0.001	-17.171	<0.001	11.668	<0.001
**RockLake**	13.402	<0.001	-16.42	<0.001	11.148	<0.001
**Brent**	23.646	<0.001	-20.768	<0.001	17.474	<0.001

Paired t-test against a hypothesised mean difference of 0 between all cut and uncut values

	t Stat	P(T<=t) one-tail
NTI (observed)	2.329	0.034
NTI (residuals)	2.329	0.034
PD (observed)	-2.225	0.038
PD (residuals)	-2.225	0.038
