# Supplementary material for: Investigating the effect of forestry on leaf-litter arthropods (Algonquin Park, Ontario, Canada)
Source: PLoS One. 2017 Jun 2;12(6):e0178568. doi: 10.1371/journal.pone.0178568 (PMC5456079; doi:10.1371/journal.pone.0178568)

**SI Supporting Information 1: Species Narratives**

Several discoveries emerged from these collections that were made possible due to the rapid publication of data prior to formal publication (Figure 1). Two of these involved the documentation of new species. The gall wasp *Aulacidea pilosellae* Kieffer (Hymenoptera: Cynipidae, BOLD:AAU8720) is a species that targets hawkweeds in the genus *Pilosella* Vaillant (Asteraceae). Native to central Europe, the first detection of this species in Canada was via the public release of barcode data for specimens collected in the trap in the cut site outside of Algonquin Park (Douglas) [[1](#_ENREF_1)]. The leafminer species *Scrobipalpula manierreorum* (Lepidoptera: Gelechiidae, BOLD:AAG9100) is a newly described species of moth that targets *Eurybia* (Aster) *macrophylla* (L.) Cassini (Asteraceae). The paratype series of this new species includes an individual collected at the Shaw Woods site as part of this work. The inclusion of this specimen in the species description was made possible by the early release of the DNA barcoding data on BOLD [[2](#_ENREF_2)].

In addition to the newly described species, there were multiple cases of morphologically cryptic genetic diversity that suggests the presence of multiple species. For example, the ant species (*Tapinoma sessile,* Dolichoderinae) is a species with a wide distribution, but characterized by deep barcode divisions within Algonquin Park (BOLD:AAA3898 and BOLD:AAM6820), suggestive of the existence of multiple species [[3](#_ENREF_3)]. The collembolan, *Parisotoma notabilis* is the most widely distributed species in Europe, but characterized by deep genetic divisions that are suggestive of cryptic species [[4](#_ENREF_4)]. We found two BINS of this species within Algonquin.

There were multiple cases (47) where species uncovered in these collections represented novel BINS to the DNA barcode library. For example, the species of Polydesmidae millipede BOLD:ADA0234 was captured at nearly all collection sites – but prior to this work was not represented in BOLD. The Hemipteran species BOLD:ABY1771 (Achilidae, BIOUG02365-E07) was, at the time of writing, a singleton BIN on BOLD. More surprisingly, based on the intense recent sampling of spiders [[5](#_ENREF_5)], we found one spider species BOLD:ACA2347 (Linyphiidae, BIOUG02613-A01) that represents an apparently new species to Canada.

**REFERENCES**

1. Moffat CE, Smith MA (2014) Pre-release detection of a biocontrol agent: combining independent and public DNA sequences to identify the first North American record of Aulacidea pilosellae (Hymenoptera: Cynipidae). The Canadian Entomologist 147: 390-395.

2. Adamski D, Landry J-F, Nazari V, Priest RJ (2014) Three new species of leaf-mining Gelechiidae (Lepidoptera) from Canada and northeastern United States. Journal of the Lepidopterists' Society 68: 101-123.

3. Smith MA, Umphrey GJ, Fisher BL, Hebert P (2005) Rapid assessment of ant diversity in a northern world heritage site using DNA barcodes (poster). First International Conference for the Barcoding of Life, Feb 6-9, The Natural History Museum London, UK: <http://www.genemetrix.net/pdf/London-AntPosterPDF.pdf>.

4. Porco D, Potapov M, Bedos A, Busmachiu G, Weiner W, et al. (2012) Cryptic Diversity in the Ubiquist Species Parisotoma notabilis (Collembola, Isotomidae): A Long-Used Chimeric Species. PLoSONE 7: e46056.

5. Blagoev GA, deWaard JR, Ratnasingham S, deWaard SL, Lu L, et al. (2016) Untangling taxonomy: a DNA barcode reference library for Canadian spiders. Molecular Ecology Resources 16: 325–341.

**Figure 1**: An example from each of the nine taxa examined here of species discovered in this work. The specific BINS and/or specimen accessions in these narratives are listed below and are all accessible via the public DOI: [dx.doi.org/10.5883/DS-ASALGONQ](http://dx.doi.org/10.5883/DS-ASALGONQ). A) BOLD:AAG9100, B) BOLD:AAU8720 , C) BOLD:AAA3898 and BOLD:AAM6820 D) BOLD:AAB2868 and BOLD:AAA4157, E) BOLD:AAU6930 (BIOUG06758-F09), F) BOLD:ADA0234, G) BOLD:AAJ2208 (BIOUG14581-A01), H) BOLD:ABY1771 (BIOUG02365-E07) and I) BOLD:ACA2347 (BIOUG02613-A01).


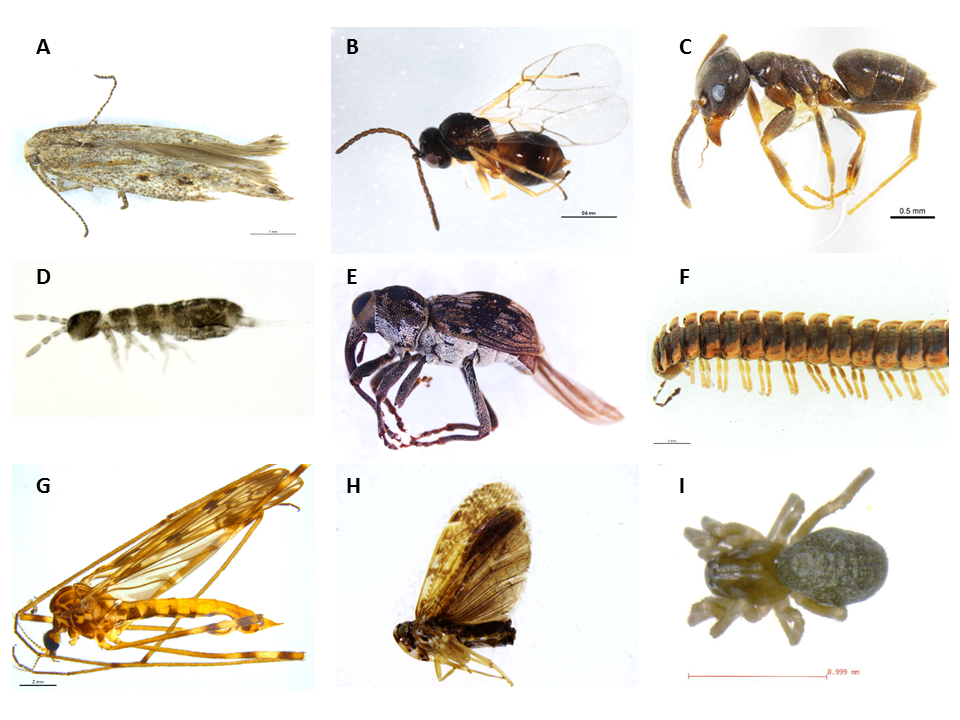

Supplement: S1 Text — The specific BINS and/or specimen accessions in these narratives are listed below and are all accessible via the public DOI: dx.doi.org/10.5883/DS-ASALGONQ. A) BOLD:AAG9100, B) BOLD:AAU8720, C) BOLD:AAA3898 and BOLD:AAM6820 D) BOLD:AAB2868 and BOLD:AAA4157, E) BOLD:AAU6930 (BIOUG06758-F09), F) BOLD:ADA0234, G) BOLD:AAJ2208 (BIOUG14581-A01), H) BOLD:ABY1771 (BIOUG02365-E07) and I) BOLD:ACA2347 (BIOUG02613-A01). (DOCX) [file pone.0178568.s007.docx]
